# Improving the quality of training paramedics by means of cadavers – a pilot study

**DOI:** 10.1186/s12909-021-02498-x

**Published:** 2021-01-21

**Authors:** Piotr Leszczyński, Bożena Muraczyńska, Arkadiusz Wejnarski, Bożena Baczewska, Maria Malm, Bartłomiej Drop

**Affiliations:** 1 Faculty of Medical Sciences and Health Sciences, University of Natural Sciences and Humanities, Siedlce, Poland; 2 Chair of Internal Medicine and Department of Internal Medicine in Nursing, Medical University of Lublin, Lublin, Poland; 3 Department of Medical Informatics and Statistics with E-learning Lab, Medical University of Lublin, Lublin, Poland

**Keywords:** Cadavers, Simulated environment, Education of paramedics

## Abstract

**Background:**

Paramedics are authorised to perform emergency procedures, including trauma assessment according to global standards. The aim of the study was to answer the question whether the use of cadavers in teaching practical competencies to medical rescue students, in the field of trauma assessment, is necessary as a supplement to learning in simulated conditions with the use of mannequins.

**Methods:**

Research included several stages. The first stage was conduction of classes for 27 students in the field of rapid trauma assessment, in accordance with the guidelines of the International Trauma Life Support. In the second stage, a plan of a test in which students had to perform an analogous procedure of rapid trauma assessment, but with the use of cadavers, human unfixed specimens, was prepared. The Delphi method was used to develop and approve checklists, as well as a scale to assess the global correctness of identification of head, torso and limb injuries by medical rescue students.

**Results:**

The identification rate was 76.54% in the head area, 67.90% in the torso area, while in the limb area it equalled 44.45%. A significant difference in scores, compared to the examination performed on a mannequin, was observed (Wilcoxon = 4.541; *p* = 0.000). The most difficult to make a correct diagnosis were injuries related to a fracture of the proximal end of the femur and a dislocated wrist (only 18.52% of correct answers). The students highly rated the usefulness of the examination, by awarding it an average of 4.76 points (SD ± 0.56) on the Likert scale (0–5).

**Conclusions:**

The study shows that the use of cadavers to teach practical competencies in the field of trauma assessment to medical rescue students can be an effective supplement to simulated learning. Students could feel the difference between the human body and the mannequin. More research is needed to assess whether realistic simulation translates into objective endpoints, such as the effectiveness of diagnosis in the examination of trauma patients. However, it should be remembered that the introduction of this teaching method is expensive and requires adequate base, as well as the compliance with a number of formal requirements.

**Supplementary Information:**

The online version contains supplementary material available at 10.1186/s12909-021-02498-x.

## Background

The profession of a paramedic authorises to perform independent emergency rescue operations at the site of an accident or disaster, especially in the field of rescuing people in a condition of immediate threat to life or health [1, 2]. As in the case of other medical professions, a paramedic is expected to have competencies in, among others, invasive procedures, such as endotracheal intubation, blood vessel cannulation, or chest puncture. In Poland, medical rescue operations that may be provided independently by a paramedic are determined by the Regulation of the Minister of Health of December 16, 2019 [3]. In order to achieve the highest level of competence, a paramedic must acquire practical knowledge in human anatomy, as well as in the area of possible changes occurring in human structures and tissues at the time of injury.

In Poland, during 3-year degree program in the field of medical rescue, practical classes including, among others, procedure patterns according to the guidelines of the European Resuscitation Council (ERC) and the International Trauma Life Support (ITLS) are conducted [4]. The dynamic development of simulation techniques in medical school and universities opens a new chapter in the education and training of paramedics in the field of clinical skills [5]. Medical simulation provides students with an authentic, realistic environment for critical thinking, in which patient safety is not compromised, and teachers with the opportunity to design scenarios that are tailored to the curriculum, thereby strengthening educational objectives [6, 7]. At the university level, simulation is a key element in the education of paramedics, providing students with a comprehensive understanding of advanced concepts in the field of anatomy, physiology, interview and physical examination, as well as performing medical rescue operations including minor surgical procedures.

The dimension of medical simulation in the area of medical rescue varies in terms of costs and technical possibilities. Cheaper versions of computer simulations (DVD, e-learning) [5, 8, 9] as well as advanced high fidelity phantoms and treatment rooms are available [10]. Live-tissue training (LTT) classes are also used to train medical staff. Working on specimens of animal origin increases the quality of training allowing the study of invasive procedures. However, evidence of high effectiveness of both simulation techniques and LTT in teaching diagnostic procedures on humans is limited [11].

Holland et al. claim that medical simulation is “*of little or no significance for surgery where work with tissues is the most important*” and that „*training on cadavers provides an opportunity to accurately understand human anatomy and helps develop competence in certain procedures*” [12]. The method that allows for the work directly on the human body without exposing the patient to danger is the use of fresh cadaver. There is evidence of the effectiveness of teaching anatomy [13, 14] as well as invasive medical procedures using cadavers [15–18]. However, this method is expensive and requires adequate base, as well as the compliance with a number of formal requirements. Therefore, it is not widely used in teaching such professions as nursing or medical rescue. Paramedics often have to act quickly and under pressure, especially where human life is at risk. The acquisition of practical competencies in e.g. invasive procedures or the ability to quickly assess injuries, using only mannequins, seems insufficient because of differences between the behaviour of human tissue and work on a mannequin.

Therefore, there is a concern that paramedics may not be properly prepared to perform certain medical procedures, as there is no requirement to work on cadavers during studies. It should be remembered that training on mannequins usually does not reflect the behaviour of human tissues under real conditions, and the professional internships of paramedics with a trauma patient prevent performing multiple examinations by a group of students.

There is still a small number of scientific reports in the literature that would relate to verification of learning outcomes among paramedics according to ITLS procedure patterns on fresh cadavers. The authors of the study tried to check the competencies of medical rescue students in the field of trauma assessment using both mannequins and cadavers. An attempt was made to determine whether the introduction of cadaver training into the curriculum of medical rescue studies is important to ensure the acquisition of practical skills in dealing with a trauma patient, compared to the medical simulation based on the use of mannequins only. To this end, an examination was conducted on the simulator and cadavers, verifying in both cases the correctness of performing specific procedures. The basic assumption of the study was to indicate whether standard teaching on mannequins is sufficient. The authors attempt to assess the quality of training paramedics with human cadavers.

## Methods

### Methods

The own research included several stages. The first stage was to conduct classes for the second and third year students (*n* = 27) in the field of rapid trauma assessment, in accordance with the guidelines of the International Trauma Life Support. The study curriculum provides teaching using training mannequins, which were used to perform a test under simulated conditions.

In the second stage, a plan of a test in which students had to perform an analogous procedure (rapid trauma assessment), but with the use of cadavers, dead human unfixed bodies, was prepared. The inclusion criterion was the completion of the dummy trauma test module by the student during the study program. None of the study participants had any previous experience of working on human cadavers. The Delphi method was used to develop and approve checklists, as well as a scale to assess the global correctness of identification of head, torso and limb injuries by medical rescue students. It belongs to the group of heuristic methods in which the knowledge, experience and opinions of experts in a given field are used to make decisions [19]. The Delphi study was conducted with the participation of teaching staff (“experts”). The experts were selected from people directly involved in teaching in the field of medical rescue and had specialist, theoretical and practical knowledge about clinical competencies required from medical rescue graduates, or paramedics. All experts had at least 5 years of experience of work in higher education. The assessment of competencies of medical rescue students using simulations was performed by a teacher on the basis of direct observation, using scenarios and checklists approved by the experts. When using checklists, the examiner selected items performed by the student. In addition, the direct observation by a teacher was supported by video recording.

Lecturers, who previously conducted classes with students, and independent teachers from outside the university (section technicians and doctors) were employed as experts. During the research, at least one university teacher and one independent expert were present at each position. This avoided bias in the assessment as a confounding factor. The checklists included a list of activities and diagnoses for the rapid trauma examination (see Additional file 1 for checklist). In order to avoid discrepancies in the assessment scale of examiners, the answer options “YES” and “NO” were used. Both the completion of the dummy and the corpse were based on the same patterns of checklists. The set of injuries has been adjusted so that they can be identified during a rapid trauma examination, in accordance with the current ITLS guidelines.

Each student underwent training in procedures and stages of dealing with cadavers. This training allowed students to get acquainted with legal, sanitary and occupational safety and health regulations, as well as gave them opportunity to refine a number of procedural skills and directly learn the differences in human anatomy. The initial training required the participants to sign declarations, committing themselves to duly respect the bodies and not to take photos and videos during the classes. The students were equipped with personal protective equipment and briefly trained in the rules of handling potentially infectious material. A basic study with the use of cadavers was conducted in 2019 in the dissecting rooms in Warsaw (Poland) with the participation of 27 Bachelor’s degree program students in the field of medical rescue. The study was approved by the bioethics committee (12/2018 UPH Siedlce), and funds were obtained from project No. 37 entitled: “Best of the Best 3.0” of the Ministry of Science and Higher Education.

### Material

Human specimens from American donors met the requirements of very restrictive procedures and were FDA approved. All specimens underwent virological tests for HIV, HBV, HCV, syphilis, and because of the low storage temperature, were biologically inactive and safe. After the donors’ death, human bodies were freshly frozen and then thawed 24 h before the start of the test preparing procedure. As a result, they achieved a natural appearance and tissue elasticity. This allowed for a proper preparation of specimens, introducing precisely determined soft and hard tissue injuries.

**Fig. 1:**
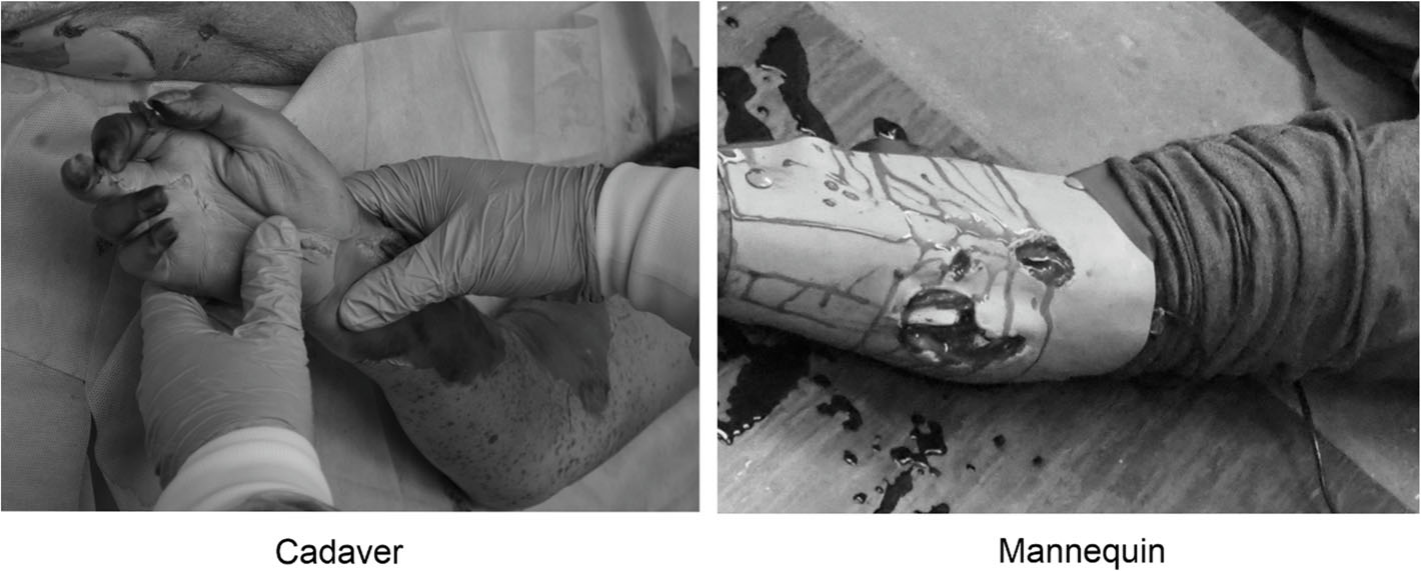
Limb Injury (Cadaver vs. Mannequin)

All participants (students) were volunteers, and signed informed consent to participate in the study. Cadavers were prepared by a team consisting of a technician, neurosurgeon and paramedic. This team introduced precise head, torso and limb injuries. The method of performing the injuries was an original method. For example, a system of overloads was used to break a limb in the right place. In a random order, students individually conducted a rapid trauma assessment of the prepared specimens (up to a maximum of 2 min) under the supervision of university teachers. The diagnosed injuries were reported by students orally, immediately after the examination of a given part of the body. Similarly to tests on a mannequin, students were not previously informed about the number and possible types of injuries. An example comparison of limb injuries simulated on mannequin and performed on a cadaver is shown in Fig. 1.

At the end of the test, students were asked to complete questionnaires, where they could assess the quality of the training and the level of comfort in performing the trauma assessment procedure using a cadaver. A 5-point Likert scale was used for the assessment, with 1 being the lowest and 5 being the highest score (see Additional file 2 for Exam Grading Survey).

### Statistical analysis

The obtained data were presented in numerical values and arithmetic means with a standard deviation. For statistical analysis, the Kolmogorov-Smirnov normality test; Spearman’s Rho correlation, the Wilcoxon test and the equivalent of the Kruskal–Wallis one-way analysis of variance were used, conducted with the use of the PAST 3.20 software. All results were considered statistically significant at *p* < 0.05.

## Results

### Characteristics of the study group

The study involved 27 medical rescue students, including 33.33% of women (*n* = 9) and 66.67% of men (*n* = 18). The average age of study participants was 22.15 years (SD ± 1.53).

### Assessment of the head injury – “HEAD” station

In the dissecting room, on a previously prepared specimen of the head, the students were required to conduct a rapid trauma assessment and identify: a foreign body in the oral cavity; bloody discharge from the ear; invagination of the skull. The average score was 76.54%. The results obtained by the students were as follows:

- identification of a foreign body in the oral cavity – 14/27 students (51.85%);
- identification of bloody discharge from the ear – 21/27 students (77.78%);
- indication of the site of invagination of the skull – 27/27 students (100.00%).

### Assessment of the torso injury – “TORSO” station

At the second stand, students conducted a rapid trauma assessment of the torso with lower extremities (up to the height of the tibiofemoral joints). They had to recognise both injuries, as well as physiological and pathophysiological mechanisms, such as: stable chest; a fracture within the acetabulofemoral joint; stable pelvis. The average score was 67.90%. The results obtained by the students were as follows:

- identification of thoracic stability – 15/27 students (55.56%);
- identification of a fracture within the acetabulofemoral joint – 19/27 students (70.37%);
- identification of pelvic stability – 21/27 students (77.78%);

### Trauma assessment of the lower and upper extremities – “LIMBS” station

**Fig. 2:**
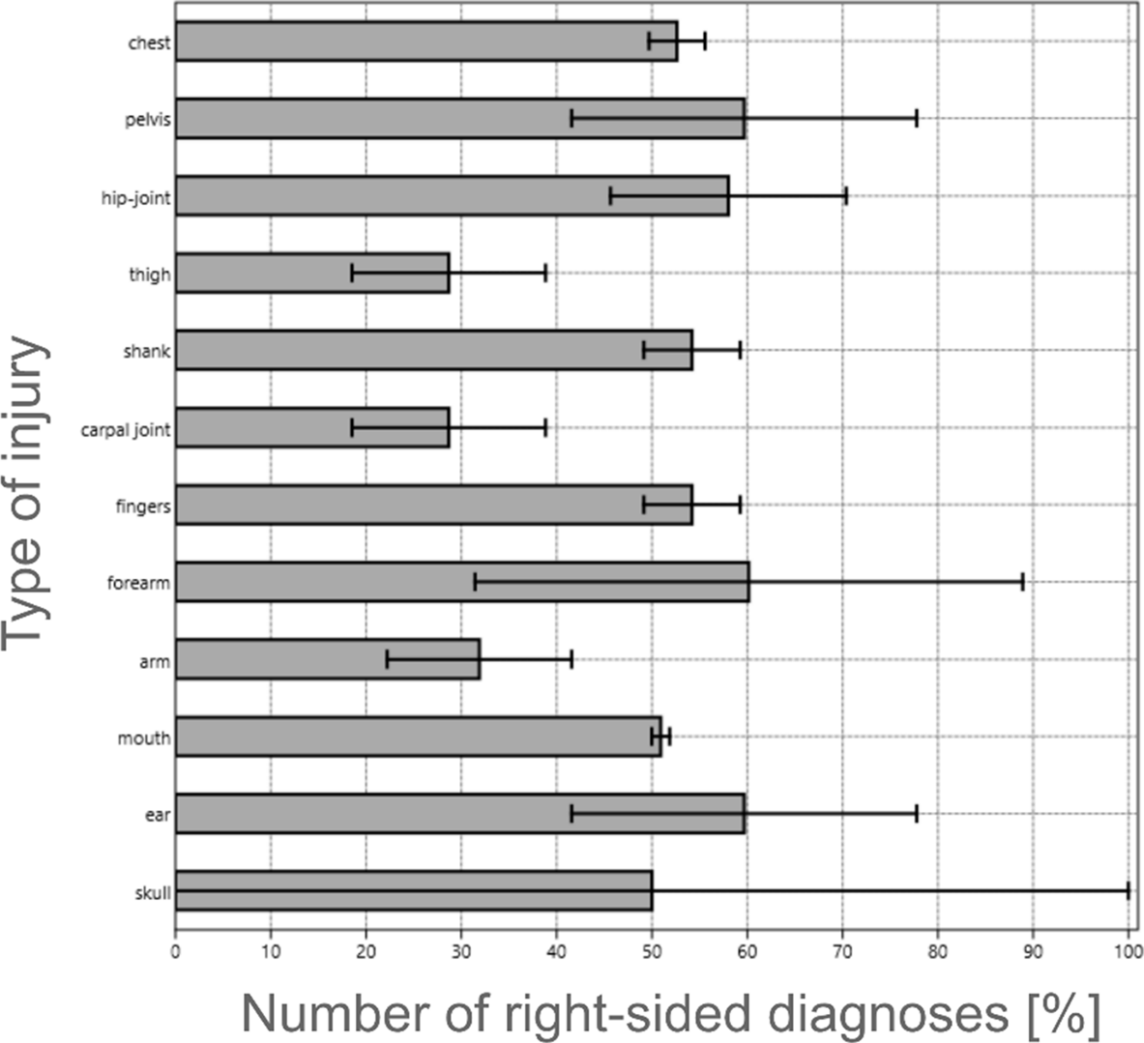
Correct diagnoses in the rapid trauma assessment

At the last stand, students were expected to examine specimens of the lower and upper extremities, which included such pathological changes as: a femoral fracture; a combined tibia/fibula fracture; a fracture of the proximal end of the humerus; a Colles’ fracture; a dislocated wrist; a phalanx fracture. The average score was 44.45%. After performing a rapid trauma assessment of the lower and upper extremities, the results obtained by the students were as follows:

- identification of a dislocated wrist – 5/27 students (18.52%);
- identification of a femoral fracture – 5/27 students (18.52%);
- identification of a fracture of the proximal end of the humerus – 6/27 students (22.22%);
- identification of a phalanx fracture – 16/27 students (59.26%);
- identification of a combined tibia/fibula fracture – 16/27 students (59.26%);
- identification a Colles’ fracture – 24/27 students (88.89%);

The average final score of the examinations of the students at all stands was: 62.96%. A detailed summary is presented in Fig. 2.

### The assessment of the examination by the students

**Fig. 3:**
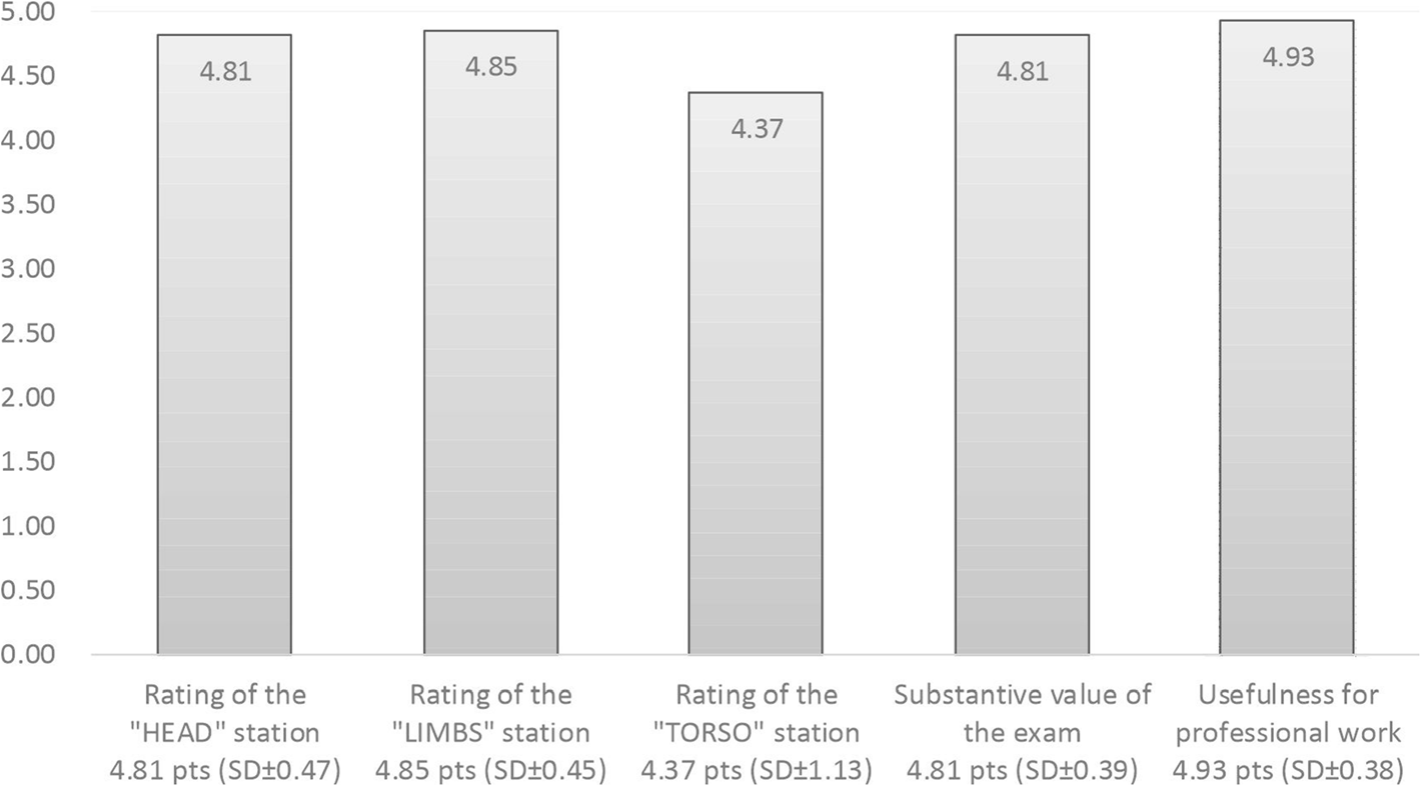
The results of the evaluation survey concerning the examination performed with the use of cadavers

At the end of the study, the students (*n* = 27) were asked to complete a short survey concerning the assessment of their satisfaction and scientific quality of the individual specimens (head, torso, extremities), their content-related value and suitability for professional work. All assessments were conducted on a Likert scale (from 0 to 5). The total average score of all survey components was 4.76 points (SD ± 0.56). A detailed summary is presented in Fig. 3.

### Statistical analysis

In order to perform statistical analyses, variable distribution normality tests were conducted. Values concerning sex did not show a normal distribution (Kolmogorov-Smirnov Test = 0.427; *p* < 0.001). Values concerning age also did not show a normal distribution (Kolmogorov-Smirnov Test = 0.279; *p* = 0.024). Therefore, nonparametric tests were used in the analysis of data covering the characteristics of the group. Based on the analysis of the number of correct responses in 12 cases of identification during the trauma assessment, no correlation with the age (rho-Spearman = − 0.154; *p* = 0.441) and sex (rho-Spearman = − 0.238; *p* = 0.232) of the students was found.

Before starting the examination, all students under simulated conditions successfully passed each element of the rapid trauma assessment, obtaining in the first test the maximum result (12/12) of correctly identified injuries for the range of examination covering 12 body parts. The variables related to the first test (on a phantom) were compared with those of the second test (on cadavers). The result obtained in the Wilcoxon test was W = 4.541 for *p* < 0.001, which confirmed statistically significant intergroup differences for the total score of examination in 12 anatomical regions. A detailed list of differences between the results in individual elements of the trauma assessment is presented in Table 1.

**Table 1:** A comparison of the results of the examination with the use of mannequins with the results of the examination with the use of cadavers

Area of trauma examination	Exam 1 (manikin)		Exam 2 (cadaver)		Wilcoxon test
	[n]^a^	[%]	[n]^a^	[%]	[p]
Cranial (skull)	27	100.00%	27	100.00%	1
Ear (external auditory canal)	27	100.00%	21	77.78%	**0.014**
Oral (mouth)	27	100.00%	14	51.85%	**< 0.001**
Arm	27	100.00%	6	22.22%	**< 0.001**
Forearm	27	100.00%	24	88.89%	0.083
Fingers	27	100.00%	16	59.26%	**< 0.001**
Carpal (wrist)	27	100.00%	5	18.52%	**< 0.001**
Crural (shin)	27	100.00%	16	59.26%	**< 0.001**
Femoral (thigh)	27	100.00%	5	18.52%	**< 0.001**
Inguinal (groin)	27	100.00%	19	70.37%	**0.005**
Pelvis	27	100.00%	21	77.78%	**0.014**
Chest	27	100.00%	15	55.56%	**< 0.001**

^a^Number of students correctly identifying the area of examination

A high score with an insignificant statistical difference in both tests was demonstrated in examination of the skull and forearm. The remaining ten injuries were definitely worse identified by students during the examination with the use of cadavers, compared to the examination with the use of a mannequin. The most difficult to make a correct diagnosis were injuries related to a fracture of the proximal end of the femur and a dislocated wrist (only 18.52% of correct answers).

The authors perceived a certain discrepancy in the survey assessment of individual stands. Therefore, a one-way analysis of variance using the Kruskal-Wallis test was performed because of the lack of normality of the variables, and the obtained result was statistically insignificant (*p* = 0.161). The correlation of variables including survey results and scores at a given stand was also analysed. In the examination of the head (rho−Spearman = 0.066; *p* = 0.745), torso (rho−Spearman = 0.336; *p* = 0.087) and lower extremities (rho−Spearman = 0.074; *p* = 0.712), no significant correlations were found.

## Discussion

In the source literature the importance of using cadavers for shaping or developing professional competencies, including practical skills necessary for the successful implementation of medical procedures in medical rescue students, is rarely discussed. Interesting results are presented in the study of Lim D. et al., which shows that training on cadavers is a desirable supplement to simulated learning and clinical internships in the profession of a paramedic [20].

The present study is the first in Europe to compare two forms of the rapid trauma assessment examination in the group of medical rescue students. Students who successfully performed all elements of the trauma assessment under simulated conditions (on mannequins) and obtained a result of 100%, had to perform them on human tissue prepared in a standardised manner. It has been proved that the teaching process using only medical simulation equipment is not sufficient. The average score of 62.96% was only slightly higher than the minimum required to succeed in the examination. When designing the study on cadavers, the authors included both injuries constituting life-threatening conditions of the patient, as well as those that are not significant in emergency procedures, especially in pre-hospital emergency care. The best result was achieved in the identification of head injuries (76.54%). The authors believe that the assessment of the continuity of bone structures (skull) is easier to identify than bone fractures surrounded by a large layer of tissue (e.g. the thigh). However, skull injuries belong to directly life-threatening conditions, therefore a positive assessment requires 100 % of correct identification in the test group. The smallest number of correct diagnoses were made during the limb examination (44.45%). Attention is drawn to the lowest recognition of injuries (18.52%) to the upper limb. In the ITLS procedure, the hands are one of the last parts of the trauma test. The authors noticed that the technique of palpating the upper limbs by the students was less accurate (e.g. using a counterweight method) than the lower limbs. Paramedics need to find life-threatening injuries quickly, and bleeding from closed upper limb fractures is less likely to cause hypovolemia than thigh fractures.

Therefore, it was demonstrated that there are statistically significant differences (*p* < 0.001) between the result of the examination performed on a mannequin and the one performed on cadavers. Before being informed about their final score, students highly rated the level of preparing and conducting the examination on cadavers in a dedicated survey, giving an average of 4.76 points (SD ± 0.56) on a scale of 0–5. The authors noticed the fact that students rated the highest the stand with limb injuries (4.85 points), at which their identification rate was the lowest. However, at no stand (head, torso, extremities), a significant correlation of variables concerning the number of correct diagnoses with the students’ survey assessment was demonstrated.

The vast majority of scientific reports confirm the high effectiveness of teaching with the use of cadavers at medical faculties [21, 22]. Although there are manual procedures that can be effectively mastered on both the cadavers and the simulator [23, 24], no solution to replace the human body in the representation of the mechanism of tissue injuries has been invented yet [25]. The authors recommend supplementing the study curriculum, especially for medical rescue students with compulsory classes performed on human unfixed specimens. Training on ITLS procedures performed on manikins is insufficient. The problem may be high costs of using cadavers in medical education [26]. Human remains decompose and do not allow for long-term use. Fixed preparations do not reflect the real plasticity of tissues. This is one of the reasons why mannequins and sham sets are used more frequently in education.

### Study limitations

The study should be considered as a pilot one, as it requires further analysis carried out on a larger group. The authors included only 27 students, excluding people studying fields other than medical rescue. In addition, no survey after the training in the medical simulation laboratory was conducted. It was decided that a comparison of participants’ satisfaction (mannequin vs. cadaver) is not the aim of the study. The experience of the authors shows that innovative classes, such as training on human specimens, are usually highly satisfying for students regardless of the subject, which would also overstate the assessment of classes on cadavers. For all students participating in the study, the initiative of training on cadavers was the first experience of a contact with a human body of key importance for their clinical role.

## Conclusions

The identification rate during a rapid trauma assessment on cadavers is significantly lower in comparison to a test performed on mannequins. The education process in the field of medical rescue in Poland requires supplementing with practical training on human specimens, so that the student can feel the difference between the human body and the mannequin. More research is needed to assess whether realistic simulation translates into objective endpoints, such as the effectiveness of diagnosis in the examination of trauma patients.

## Supplementary Information

**Additional file 1.** Checklist.

**Additional file 2.** Exam Grading Survey.

## Data Availability

The data that support the findings of this study are available on request from the corresponding author. The data are not publicly available due to privacy or ethical restrictions.

## Abbreviations

ERC: European Resuscitation Council
ITLS: International Trauma Life Support
LTT: Live-tissue training
