# Supplementary material for: Improving the quality of training paramedics by means of cadavers – a pilot study
Source: BMC Med Educ. 2021 Jan 21;21:67. doi: 10.1186/s12909-021-02498-x (PMC7836173; doi:10.1186/s12909-021-02498-x)
Supplement: Supplementary file 2 — Additional file 2. Exam Grading Survey. [file 12909_2021_2498_MOESM2_ESM.docx]

|  | Exam Grading Survey | | | | | |
| --- | --- | --- | --- | --- | --- | --- |
|  | Participant No ………………………………. | |  |  |  |  |
|  |  |  |  |  |  |  |
|  | Rate (on a scale of 1 to 5) satisfaction with participation in: | | | | | |
|  | (mark the selected field with a cross) | | | | | |
|  |  | 1 | 2 | 3 | 4 | 5 |
| 1 | Rating of the "HEAD" station |  |  |  |  |  |
| 2 | Rating of the "LIMBS" station |  |  |  |  |  |
| 3 | Rating of the "TORSO" station |  |  |  |  |  |
| 4 | Substantive value of the exam |  |  |  |  |  |
| 5 | Usefulness for professional work |  |  |  |  |  |
|  |  |  |  |  |  |  |
